# Towards the Personalized Treatment of Glioblastoma: Integrating Patient-Specific Clinical Data in a Continuous Mechanical Model

**DOI:** 10.1371/journal.pone.0132887

**Published:** 2015-07-17

**Authors:** Maria Cristina Colombo, Chiara Giverso, Elena Faggiano, Carlo Boffano, Francesco Acerbi, Pasquale Ciarletta

**Affiliations:** 1 MOX—Department of Mathematics, Politecnico di Milano, Piazza Leonardo da Vinci 32, 20133 Milano, Italy; 2 Fondazione CEN, Piazza Leonardo da Vinci 32, 20133 Milano, Italy; 3 Labs—Department of Chemistry, Materials and Chemical Engineering, Politecnico di Milano, Piazza Leonardo da Vinci 32, 20133 Milano, Italy; 4 Neuroradiology—Fondazione I.R.C.C.S. Istituto Neurologico Carlo Besta, Via Celoria 11, 20133 Milano, Italy; 5 Department of Neurosurgery—Fondazione I.R.C.C.S. Istituto Neurologico Carlo Besta, Via Celoria 11, 20133 Milano, Italy; 6 Sorbonne Universités, UPMC Univ Paris 06, CNRS, UMR 7190, Institut Jean Le Rond d’Alembert, F-75005 Paris, France; University of Pécs Medical School, HUNGARY

## Abstract

Glioblastoma multiforme (GBM) is the most aggressive and malignant among brain tumors. In addition to uncontrolled proliferation and genetic instability, GBM is characterized by a diffuse infiltration, developing long protrusions that penetrate deeply along the fibers of the white matter. These features, combined with the underestimation of the invading GBM area by available imaging techniques, make a definitive treatment of GBM particularly difficult. A multidisciplinary approach combining mathematical, clinical and radiological data has the potential to foster our understanding of GBM evolution in every single patient throughout his/her oncological history, in order to target therapeutic weapons in a patient-specific manner. In this work, we propose a continuous mechanical model and we perform numerical simulations of GBM invasion combining the main mechano-biological characteristics of GBM with the micro-structural information extracted from radiological images, i.e. by elaborating patient-specific Diffusion Tensor Imaging (DTI) data. The numerical simulations highlight the influence of the different biological parameters on tumor progression and they demonstrate the fundamental importance of including anisotropic and heterogeneous patient-specific DTI data in order to obtain a more accurate prediction of GBM evolution. The results of the proposed mathematical model have the potential to provide a relevant benefit for clinicians involved in the treatment of this particularly aggressive disease and, more importantly, they might drive progress towards improving tumor control and patient’s prognosis.

## Introduction

Malignant brain tumors are among the most aggressive and lethal forms of cancer, with and estimated prevalence of 138,054 cases in 2010 in the United States [1]. Malignant gliomas (MG), which are derived from transformed glial cells, represent almost 80% of primary brain tumors, with an incidence of 4.11 new cases every 100,000 inhabitants in adult population, that increases two to four times in people from the sixth to eighth decades of life [2]. Glioblastoma multiforme (GBM), World Health Organization grade IV [3], is the most common and biologically aggressive type of MG. Characteristic features of GBM are uncontrolled cellular proliferation, diffuse infiltration and invasion, necrosis, angiogenesis and genetic instability. These conditions, together with a putative role of a subpopulation of Cancer Stem Cells (CSCs), make a definitive treatment particularly difficult. In particular, the infiltrated nature of tumoral glial cells, with difficulty in distinguishing intraoperatively the viable tumor tissue at the margin of the resection [4], makes a complete surgical resection feasible only in a low percentage of cases [5, 6]. Some advances in technology, in particular the use of fluorophore like 5-ALA [7] or fluorescein [8, 9], or intraoperative MRI [10] have led to an increase in resection percentage. However, GBM still harbors a very poor prognosis with a median survival of only 18 months even when maximal therapy consisting of complete surgical removal, radiotherapy (RT) and chemotherapy (CHT) is performed [11]. In fact, GBM almost invariably recurs at the margin of the resection cavity, independently from the post-operative treatment administered. The biological characteristics of the GBM together with the tight relationship of the tumor with eloquent areas of the brain make it difficult to develop aggressive local therapies that could theoretically allow a better local tumor control. In addition, the impossibility to predict the areas where tumor cells will regrow after treatment is one of the factors that limit the chance of targeting the therapies toward these areas immediately at the beginning of the clinical history and during disease progression. A multidisciplinary approach including new strategies of radiological diagnosis associated with mathematical modeling of tumor growth in a patient-specific manner would probably allow a better definition of the therapeutic options in every single patient with GBM, with a possible impact on tumor control and survival. Indeed, biomathematical modeling could be helpful to clinicians in developing therapeutic strategies as it potentially offers a predictive tool for investigating the dynamics of cancer formation and evolution. In particular, the ultimate goal of biomathematics for cancer is the identification of the most suitable theoretical models and simulation tools, both to describe the biological complexity of carcinogenesis and to predict tumor evolution, in order to improve therapeutic strategies and, ultimately, patients’ quality of life. Therefore, during the last decades, the capability of tumor to grow and invade the surrounding tissue has gained the attention of the mathematical and the physical research communities and numerous mathematical models have been proposed. Without loss of general characteristics, GBM growth models can be classified into three categories, based on their observation scale [12, 13]: *cellular and microscopic models* (discrete models), that describe the behavior of individual cells and eventually the interactions between cells and their environment [14, 15]; *hybrid discrete-continuous models* [16, 17], in which a continuous deterministic model is coupled with a discrete cellular automata-like approach and, finally, *macroscopic* (continuous) *models* [13, 17, 18, 19, 20, 21], in which tissue level processes are described by macroscopic averaged quantities, *e.g*. volumes, densities or flows. A more exhaustive description on mathematical modeling in tumor research is reported by the extensive reviews [22, 23, 24, 25].

The most widely used continuous GBM models are reaction-diffusion models, encapsulating a simple diffusion-reaction equation for the tumor cells [19, 26, 27]. These diffusive models can eventually account for the *heterogeneity* of the brain tissue thanks to a space-dependent glioma diffusion coefficient [20, 28, 29], whose value in the white and in the grey matter can be estimated using in vivo post-contrast T1- weighted and T2-weighted MRI data [30, 31]. These reaction-diffusion models, despite their simplicity, have been applied also for predicting survival of individual patients following resection or other treatments, such as RT or CHT [21, 30, 31, 32, 33, 34].

Some efforts to include the anisotropic motion of cells, which have been shown to play an important role in brain tumor invasion [35, 36], can be found in the reaction-diffusion GBM model proposed in [37], where the cancer cell diffusion tensor was estimated using the diffusion tensor imaging (DTI), an imaging technique introduced in the early 90s [38]. DTI is based on Diffusion Weighted Magnetic Resonance Imaging (DW MRI), which measures the magnitude of water diffusion in biological tissues and provides indirect information on fibers structure, since the random brownian motion of water molecules is highly restricted by the surrounding geometry. In the DW images, the local magnitude of water diffusion along a specific direction is described by an apparent diffusion coefficient. For anisotropic tissues, such as white matter, a single coefficient is not sufficient to describe the whole diffusive process and at least six independent components are required, which are encapsulated in the symmetric *diffusion tensor* **D**. Therefore, DTI is nowadays the only non-invasive method for characterizing the micro-structural architecture of the brain bundles, for deriving the preferential direction of water diffusion and, at the same time, of cell migration. Indeed, Deisboeck et al. [35, 39] experimentally proved that also the motion of glioma cells, as the one of water molecules, follows white matter fiber tracts.

Despite providing the preferential direction of cell migration, DTI does not give a direct measurement of the extent of cell motion and growth along the fiber paths, which is regulated by different chemical and mechanical cues [40, 41, 42]. Since the interaction of tumor cells with white matter fiber bundles is far more complex than simple water diffusion, a pure reaction-diffusion model such as the one proposed in [30, 32, 37] cannot take into account the generation and accumulation of forces occurring between the host and the malignant tissue and within the tumor itself [43].

Mechanical and biochemical interactions occurring inside the tumor cells and between the solid tumor and the external environment can be easily incorporated in discrete/hybrid models and in continuous mechanical models. In particular, at the cellular scale, notable examples can be found in the discrete patient-specific agent-based glioma model proposed by Chen et al. [14] and in the hybrid model defined in [16] by the coupling of a cellular automaton model for brain tumor growth and the diffusion of nutrients. Even if the limitation in the number of entities (and thus in the tumor dimension) that can be simulated by a discrete/hybrid model might be circumvented considering that a single voxel represents several thousand cells [14], a continuous representation of the tumor evolution might still be preferable, since it allows modeling with low computational costs the temporal and spatial macro-scale evolution of the tumour, which is the key feature required in clinical practice.

Indeed, continuous mechanical models and multiphase models [44, 45], based on the theory of mixture [46], seem more suitable to correctly describe tumor growth process at a macroscopic scale, as they incorporate the mass, momentum and energy balances that drive the system evolution [47, 48, 49, 50]. Even though some recent attempts to include mechanical balance laws into the mathematical description of GBM growth and evolution have been done [13, 51, 52], patient-specif heterogeneous and anisotropic data in continuous mechanical models have never been considered.

Therefore, in the present paper, starting from the work done in [47, 53], we propose and numerically simulate a patient-specific mechanical model of glioblastoma tumor growth with diffuse interface. The model is derived considering the mass and momentum balance of a binary mixture composed by tumor cells and healthy environment (including interstitial liquid and healthy cells) and it consists of a fourth order non-linear advection-reaction-diffusion equation for the tumor phase coupled with a reaction-diffusion equation for the nutrients. Enforcing thermodynamic consistency, the model takes into account the viscous interactions among the phases and the mechanical interactions responsible of cell-cell and cell-matrix adhesion forces. An interesting aspect of the proposed approach is the introduction of the directed motion of tumor cells towards increasing gradient of nutrients (i.e. chemotaxis) and along fibers path, that leads to the definition of a modified chemotactic flux [54]. In this way, patient-specific heterogeneous and anisotropic DTI data not only define the components of the *diffusion tensor* **D** representing nutrients’ diffusion, but they also describe the *tensor of preferential directions*, **T** used to describe the local cell motility in response to the diffusing nutrients [54]. Consequently, the model is not only capable of describing the different advective and diffusive behavior of cancer cells into the white and gray matter, but it also directly represents the active motion of cells along preferential directions in response to nutrients’ concentration.

## Materials and Methods

### Collection of Clinical Data

#### Image acquisition

Imaging data of a patient with a right parietal GBM were acquired in the context of normal clinical practice at the Fondazione IRCCS Istituto Neurologico Besta by using a 3T Magnetic Resonance (MR) imaging scanner (Achieva; Philips Healthcare) equipped with a 32-channel phased array coil. Clinical imaging sequences included pre- and post-contrast axial volumetric T1 spin echo (SE) sequences, axial volumetric T2-turbo spin echo (T2-TSE) and a sagittal volumetric fluid attenuated inversion recovery (FLAIR). Pre-contrast whole-brain DTI data-sets were acquired using a single shot spin-echo echo planar imaging (EPI) sequence (TR shortest (4687 ms), TE 80 ms, voxel 2.20×2.20×2.20 (mm^3^), slices 90, SENSE 2, FAT SAT SPIR 200 Hz). The DTI protocol was multi-shell. Diffusion gradient encoding was applied in 44 noncollinear directions with maximum b-value = 1100 smm^−2^, in 12 noncollinear directions with b-values = 50, 250, 350, 600, 800 smm^−2^ and 3 noncollinear directions with b-value = 0 smm^−2^ (107 imaging volumes total). The patient signed a written consent to the MRI test in the context of normal clinical practice, including clinical researches. The patient was not submitted to any specific procedure different from normal clinical practice and the collected patient data was anonymized and de-identified prior to analysis, so that no specific approval by Ethical Committee was considered necessary. Anonymization was performed by the neuroradiology unit of the Besta Neurological Institute, independently from the researchers involved in the paper. Furthermore, the authors involved in this study did not act as treating doctors for the clinical case from which the neurological images were taken.

#### Data processing

Diffusion data were processed using a comprehensive correction pipeline with TORTOISE [55]. T2-TSE images were used as the structural target for DTI data processing. T2-TSE image were aligned to the hemispheric mid line and the anterior and posterior commissure planes using MIPAV [56]. DTI dataset were corrected to reduce the effects of rigid body motion, eddy current distortions [57], and EPI distortions [58]. Corrections were performed in the native space, and appropriate rotations will be applied to the b-matrix [57, 59]. Then, robust estimation of tensors by outlier rejection (RESTORE) [60] were used to estimate the diffusion tensor and tensor derived metrics. The RESTORE algorithm have been selected for its ability to detect and remove artifactual data points on a voxel-wise basis, correcting for subtle artifacts such as cardiac pulsation and respiration signal drop-outs, which has been shown to be an important consideration in clinical analyses of DTI data [61].

### The Mathematical Model

The tumor lesion and the surrounding environment are described though incompressible binary mixture model, composed by a cellular phase of proliferating cancerous cells, with volume fraction *ϕ*_c_ and a liquid phase, with volume fraction *ϕ*_ℓ_, modeling the host cells, the extracellular matrix, the interstitial fluid environment and necrotic cells. Assuming that these two phases fill all the available space, the saturation relation *ϕ*_c_ + *ϕ*_ℓ_ = 1 holds.

We consider a bounded domain Ω ∈ ℝ^3^ representing the whole brain, with boundary ∂Ω, and a time period [0, *T*], *T* < ∞, representing the time interval in which the tumor is evolving. We define the tumor region Ω_*t*_(*t*) = {**x** ∈ Ω : *ϕ*_*c*_(**x**, *t*) ≥ *ɛ*_*t*_}, with *ɛ*_*t*_ > 0, and the healthy host tissue region Ω_*h*_(*t*) = Ω\Ω_*t*_(*t*). The two regions Ω_*t*_(*t*) and Ω_*h*_(*t*) evolve in time, accordingly to the dynamics of the cellular phase. We associate a convective velocity **v**_*i*_, *i* = {*c*, ℓ}, to each phase and we treat the cellular and the water phases as incompressible fluids whose *true mass densities* [46] are constant and equal to water density *γ*. The mathematical model is obtained defining the mass balances for both phases

$$\begin{array}{c} \gamma \;[\frac{\partial \phi_{i}}{\partial t}+\nabla \cdot \left(\phi_{i}v_{i}\right)]=\Gamma_{i}+\nabla \cdot K_{i},\;\text{with}\;\;i=\left\{c,\ell \right\}. \end{array}$$ (1)

In Eq (1), Γ_i_ and *K*_*i*_ represent the volumetric source of mass production/loss and the non-convective mass flux of the *i*-phase, respectively. Since the mixture is closed, we impose Γ_c_ = −Γ_ℓ_ and *K*_*c*_ = −*K*_ℓ_ in order to guarantee the conservation of mass and flux exchanged among the phases. For instance, the liquid phase contains both dead cells and healthy living cells: when a cancerous cell dies, it becomes part of the liquid phase and, vice versa.

Since growth processes and mass transport phenomena in living materials are driven by the local concentration of nutrients and growth factors, we introduce proper constitutive equations for Γ_*i*_ and *K*_*i*_ based on nutrient availability. We consider oxygen as the main nutrient source for tumor cells and, defining *n* its concentration and *ρ*_c_ = *γϕ*_*c*_ the *apparent cell mass density* [46], we model the net cell proliferation rate Γ_*c*_ with

$$\begin{array}{c} \frac{\Gamma_{c}}{\gamma }=\nu_{c}\frac{\rho_{c}}{\gamma }\;(\frac{n}{n_{s}}-\delta_{c})\;(1-\phi_{c})=\nu_{c}\phi_{c}\;(\frac{n}{n_{s}}-\delta_{c})\;(1-\phi_{c}). \end{array}$$

In the above equation, *ν*_*c*_ is the cancer cell proliferation rate, *n*_*s*_ is the physiological concentration of oxygen inside the tissue and *δ*_*c*_ is the rate of apoptosis in hypoxic conditions. The factor (1 − *ϕ*_*c*_) mimics the decrease of the cellular proliferation rate due to contact inhibition, as the tumor approaches the saturation condition.

Furthermore, the mass flux *K*_*c*_, which represents the chemotactic movements up to an increasing gradient of nutrients, is expressed by

$$\begin{array}{c} K_{c}=-k_{n}\rho_{c}T\nabla n, \end{array}$$ (2)

where *k*_*n*_ is the chemotactic coefficient and **T** is a tensor defining the alignment of fibers. The expression in Eq (2) has the same form of the chemotactic term introduced by [54] and widely used in mathematical models of cell motion [62, 63]. Here, we modify the original Keller-Segel model [54] including the tensor **T** into the original expression, so that we are able to model the biased motion of cells along fibers. The introduction of **T** in the chemotactic term is particularly important in tumors growing in an highly heterogeneous environment, such as the preferential paths of GBM cell motion along the white matter fibers. In other words, *K*_*c*_ is able to describe, at the same time, both the directional motion of glial cells in response to nutrient concentration and their tendency to anisotropically move along the white matter fibers.

In order to close the equation system, it is necessary to define proper laws for the convective velocities **v**_*c*_ and **v**_ℓ_, appearing in Eq (1). Following the work done in [53], we make use of a thermodynamically-consistent approach, modeling the viscous interactions and mechanical forces resulting from the cells’ ability to adhere to each other or to the extracellular matrix, through adhesion molecules called CAMs located at the cellular membrane [64]. Thus, we define a Helmholtz free energy which takes into account both local and long-range interactions among the components and we assume that the energy dissipation in the system is due only to the viscous interactions between the phases. Then, we use Rayleigh’s variational principle to derive the system dynamics, minimizing the Rayleighian with respect to **v**_*c*_ and **v**_ℓ_ as in [65]. Thus, we obtain the following relation between the convective velocities:

$$\begin{array}{c} v_{c}-v_{l}=-K\left(\phi_{c}\right)\nabla \;(f\left(\phi_{c}\right)-\epsilon^{2}\Delta \phi_{c})\;. \end{array}$$ (3)

**Fig 1:**
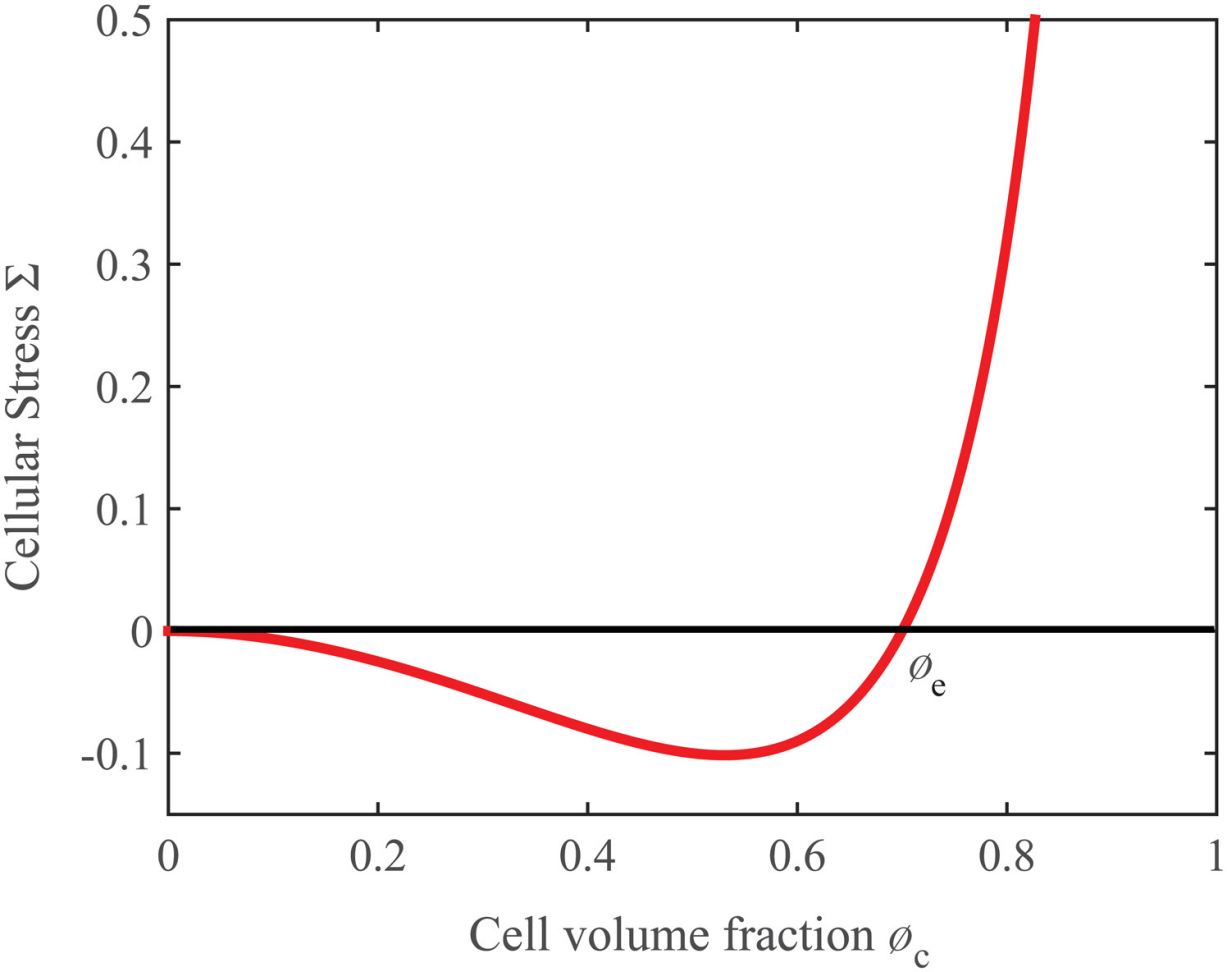
Excesses stress Σ exerted by the cells in the case of homogeneous tissue, i.e. ∇*ϕ*_c_ = 0. For physical and biological consistency, when *ϕ*_*c*_ < *ϕ*_*e*_ cells experience an adhesive force (*f*(*ϕ*_*c*_) < 0), whereas for *ϕ*_*c*_ > *ϕ*_*e*_, as cells are very close, a repulsive force acts among them and *f*(*ϕ*_*c*_) > 0. The threshold value *ϕ*_*e*_ is called state of mechanical equilibrium. The repulsive force becomes infinite in the limit that cells fill the whole volume.

In Eq (3), the motility coefficient $K(\phi_{\text{c}})=\frac{{\left(1-\phi_{\text{c}}\right)}^{2}}{M}$ is related to the inverse of the friction parameter *M*, *f*(*ϕ*_c_) is the derivative of the bulk free-energy per unit of volume, ψ, with respect to the cellular volume fraction, i.e. *f*(*ϕ*_c_) = ∂ψ/∂*ϕ*_c_, and the term in *ϵ* ^2^ represents a surface potential energy penalizing large gradients of cellular volume fraction [47]. If we call Σ = *f*(*ϕ*_c_) − ϵ^2^Δ*ϕ*_*c*_ the excess of pressure exerted by the cells and we assume that no external forces act on the highly viscous mixture, the center of mass does not move and the velocity of the cellular phase can be expressed by a Darcy-like law **v**_*c*_ = −*K*(*ϕ*_c_)∇Σ. A proper expression for *f*(*ϕ*_c_) can be empirically defined considering that, for physical and biological consistency, the cell-cell interaction should be attractive within a certain low range of cell density and repulsive at higher values. Therefore, it is possible to mathematically define a threshold value *ϕ*_*e*_, called *state of natural equilibrium* [45], for which *f*(*ϕ*_*e*_) = 0 and no excess pressure is exerted on neighbors, whereas for *ϕ*_*c*_ < *ϕ*_*e*_ cells are attracted to each other, i.e. *f*(*ϕ*_c_) < 0, and for *ϕ*_*c*_ > *ϕ*_*e*_, cells experience a repulsive force, i.e. *f*(*ϕ*_c_) > 0. Therefore, a suitable form of *f*(*ϕ*_c_) is [45, 48, 53]

$$f\left(\phi_{c}\right)=E\frac{\phi_{c}{}^{2}(\phi_{c}-\phi_{e})}{1-\phi_{c}},$$ (4)

where *E* is the Young’s Modulus of the brain matter, as sketched in Fig 1.

Finally, we propose a time dependent diffusion-reaction equation for the nutrient concentration. We assume that the vasculature is homogeneously distributed in the whole domain and we do not take into account the angiogenesis, i.e. the formation of new blood vessels. In this situation, tumor cells receive oxygen and growth factors only via diffusion inside the brain tissue. We assume also that the net nutrient uptake in the healthy tissue and in the fluid region is negligible compared to the uptake in the tumoral environment and, whenever oxygen is consumed by the host cells, it is instantaneously replaced by the normal vasculature supply. On the contrary, the cellular uptake generally exceeds the supply in the tumor region. Thus, calling *δ*_*n*_ the rate of consumption of nutrients by tumor cells, *S*_*n*_ the nutrient transfer rate between blood and tissue, the evolution in space and time of the nutrient concentration can be described by the following partial differential equation

$$\begin{array}{c} \frac{\partial n}{\partial t}=\nabla \cdot \left(D\;\nabla n\right)+S_{n}\left(n_{s}-n\right)-\delta_{n}\phi_{c}n\;, \end{array}$$ (5)

that, substituting the mass balance of the cellular phase, simplifies as

$$\begin{array}{ccc} \frac{\partial \phi }{\partial t} & = & \nabla \cdot (\frac{\phi {\left(1-\phi \right)}^{2}}{M}\nabla \left(f\left(\phi \right)-\epsilon^{2}\Delta \phi \right))+\\ & + & \nu \phi \left(\frac{n}{n_{s}}-\delta \right)\left(1-\phi \right)-\nabla \cdot (k_{n}T\phi \;\nabla n)\;. \end{array}$$ (6)

For the sake of simplicity, hereafter we drop the subscript *c* to denote the cellular tumor fraction.

The system of Eqs (5) and (6) allows to determine the evolution of the unknown fields *ϕ*(**x**, *t*) and *n*(**x**, *t*), ∀ **x** ∈ Ω and ∀ *t* ∈ [0, *T*], if proper initial and boundary conditions are provided. GBM differs from many solid tumors because it is characterized by a smooth gradient of tumor cell density instead of presenting a sharp interface at the host/tumor boundary. Thus, it seems reasonable to hypothesize that *ϕ*(**x**, 0) = *ϕ*_0_(**x**) follows a normal smooth distribution in space with a maximum slightly higher than *ϕ*_*e*_ reached in the center of the tumor. In order to obtain the initial oxygen concentration *n*(**x**, 0) = *n*_0_(**x**), we solve the steady version of the nutrient governing Eq (5), corresponding to the initial cellular distribution, *ϕ*_0_. The solution obtained is equal to *n*_*s*_ outside the tumor area and decreases getting closer to the core of the glioblastoma, in accordance with the increase of *ϕ*_0_ in this area.

Finally, it is mandatory to define boundary conditions for the governing equations. We impose a null Dirichlet condition and a null Neumann condition for the cell volume fraction at the boundary of the cranial skull:

ϕ=0,on∂Ω,∀t∈[0,T] (7)

$$\nabla \phi \cdot \hat{n}=0,\;\text{on}\;\partial \Omega ,\forall \;t\in [0,T]$$ (8)

where $\hat{\text{n}}$ is the outward boundary normal. For the nutrients, we impose the Dirichlet condition

$$\forall \;t\in \left[0,T\right]\;n=n_{s},\;\text{on}\;\partial \Omega ,$$ (9)

since we suppose that the brain boundary is far enough from the tumor location and consequently the oxygen concentration is maintained equal to the physiological value by the vasculature.

### Numerical Implementation

#### Mesh Generation

**Fig 2:**
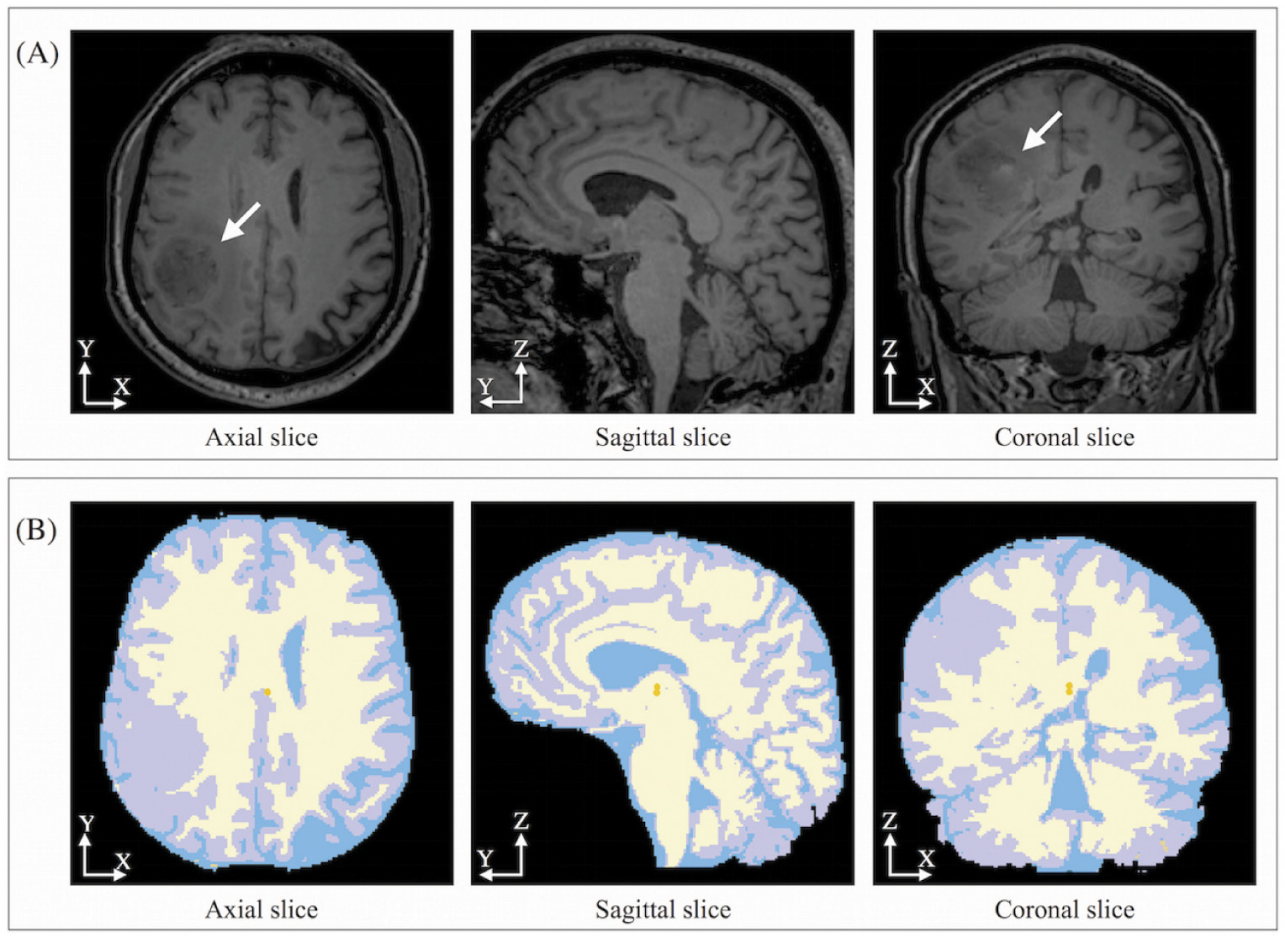
Post-contrast T1-MR of a patient affected by GBM and corresponding segmented slices. (A) Axial, sagittal and coronal slices of post-contrast T1-MR in a patient with right parietal GBM (white arrow), used for image segmentation. (B) In the segmented brain image, the white region represents the white matter, the grey areas indicate the grey matter, while the cerebrospinal fluid is labeled by the blue color.

The creation of computational grids able to reproduce the patient-specific brain geometry without exceeding in the computational costs is a challenging task. The first step to generate a patient-specific computational mesh is the medical image segmentation, which is the process of identifying and labeling regions of interest within an image. To generate the anatomical mesh of the brain and tumor we use the post-contrast T1-MR sequence (Fig 2(A)). Using an expectation maximization approach [66] implemented in the open source software package 3D Slicer [67], the anatomical structures are automatically segmented and the four areas of interest (i.e. gray matter, white matter, cerebrospinal (CSF) fluid and background) are identified and labeled. The segmented image obtained is depicted in Fig 2(B). Once the brain segmentation is done, we manually segment the GBM region, since voxels occupied by the tumor have an intensity comparable to ones occupied by grey matter an automatic process cannot be implemented.

After the creation of the brain labeled map and the identification of the tumor region, the computational mesh is obtained first by extracting the external brain surface using the marching cube algorithm, then operating a Taubin surface smoothing and a uniform mesh refinement with the scripts implemented in The Vascular Modeling Toolkit (www.vmtk.org) [68]. Thirdly, we build the tetrahedral mesh using the TetGen library [69]. The mesh is then refined near the area of interest (e.g. in the region in which the tumor grows) in order to control the numerical error without exceeding in computational costs. After the mesh refinement, we assign the information contained in the labeled map to the computational grid in order to obtain a labeled mesh. This procedure is implemented in Python using the Visualization Toolkit (www.vtk.org) library.

#### Finite Element discretization

Once the brain mesh is created, it is possible to proceed to the spatio-temporal discretization of the system Eqs (5) and (6). In particular, we perform a spatial discretization with linear tetrahedron *P*_1_ elements and a time discretization with the Crank-Nicholson algorithm [70]. Once the equations are discretized, they can be easily implemented using the open source software FEniCS [71], using Python as programming language. The only required mathematical trick is to rephrase the fourth-order Eq (6) as the following second-order equations

$$\frac{\partial \phi }{\partial t}-\nabla \cdot \left(\phi K(\phi )\nabla \Sigma \right)-\nu \phi (\frac{n}{n_{s}}-\delta )(1-\phi )+\nabla \cdot \left(k_{n}\phi T\nabla n\right)=0$$ (10)

$$\Sigma =f(\phi )-\epsilon^{2}\Delta \phi .$$ (11)

One of the main advantage of using FEniCS as computational resource is that it offers built-in classes and an automatic approach to nonlinear variational problems. Furthermore FEniCS allows the introduction of real patient-specific data, taken from the medical images, as discussed in the next subsection.

#### Reconstruction of patient-specific data and parameters’ estimation

**Fig 3:**
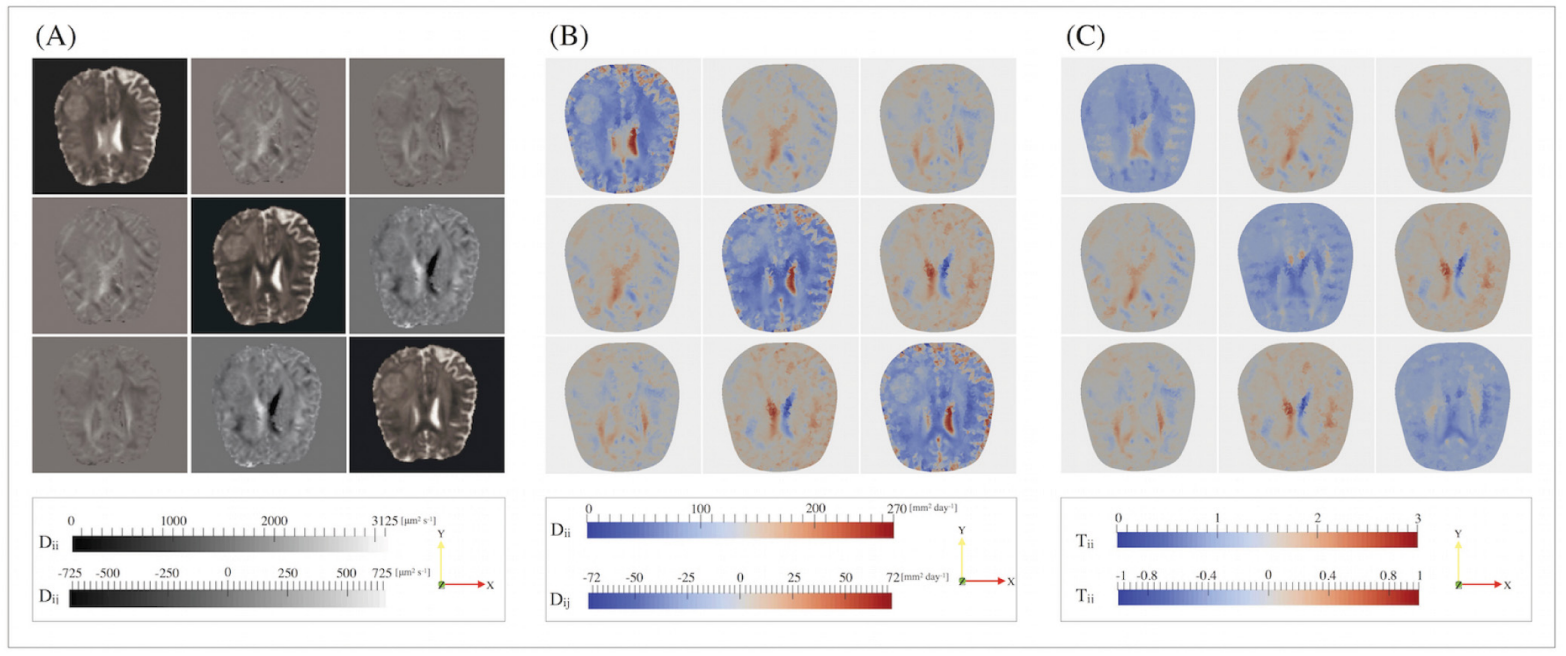
Patient-specific medical and numerical DTI data, depicted on a slice cut along the plane xy. (A) A single component of the tensor **D**, obtained from the DTI medical images, is represented for each image: the intensity of the voxels is related to the diffusion coefficient along the relative direction (see the gray-scale at the bottom). (B) Numerical patient-specific components of the *diffusion tensor* **D** depicted on the same slice of the medical images: the diffusion coefficient is higher in the region occupied by the cerebrospinal fluid (red colored areas), where the diffusion is unconstrained. (C) Corresponding patient-specific components of the *tensor of preferential directions* **T**: in isotropic region, e.g. the cerebrospinal fluid and the grey matter, *T*_*xx*_ ≈ *T*_*yy*_ ≈ *T*_*zz*_ ≈ 1 and *T*_*xy*_ ≈ *T*_*xz*_ ≈ *T*_*yz*_ ≈ 0, while in the white matter, instead, 0 < *T*_*ii*_ < 3 with i = (x, y, z) and 0 < *T*_*ij*_ < 1 with i, j = (x, y, z) and *i* ≠ *j*, denoting an anisotropic region.

The clinical usefulness of mathematical models mainly relies on the identification of the correct biological parameters to be included in the model. Indeed, a model is potentially predictive if all parameters are measured or estimated from specific biological experiments on the system under study. Furthermore, as the evolution of a tumor can be significantly affected by the different environmental conditions, the possibility to specify the mathematical model on a single patient, through the introduction of patient-specific data, is a mandatory request for a clinical use. In principle, all the parameters appearing in Eqs (5) and (6) can be either estimated from *in-vitro* and *in-vivo* biological experiments or extracted from clinical exams, as for **D** and **T**, whose components can be obtained from the DTI images of the patient. In particular, assuming that the oxygen diffuses coherently with the water molecules, its behavior can be described by the water diffusion and thus the nutrients’ diffusion tensor **D**, appearing in Eq (5), can be directly obtained from the DTI measurements. Being the tensor **D** symmetric, i.e. *D*_*ij*_ = *D*_*ji*_, all the necessary information on the diffusion coefficients is provided by the six DTI-maps in greyscale, each of which represents a component of the diffusion tensor. In Fig 3(A) we report, as an illustrative example, the components of the tensor **D** on a slice along the *xy*-plane in the middle point of the *z*-axis of the brain, as they are obtained from the DTI medical examination. In Fig 3(A), brighter voxels (e.g. the ones in the ventricles area) correspond to higher diffusion values, while darker ones represent lower values of the corresponding *D*_*ij*_ component. Once the six DTI images are registered with the T1-MRI image used for creating the computational mesh [67], we associate each value of a specific voxel in the DTI image to the tetrahedron which occupies the same location of the voxel in the computational mesh. The resulting data, which are reported in Fig 3(B) for the same slices considered in Fig 3(A), are then simply included in the model thanks to a specific FEniCS function. Besides, supposing that cells can chemotactically move along the same fiber paths of water diffusion, the tensor of the preferential directions **T** can be obtained from the same DTI maps, defining each component as

$$T_{ij}=\frac{D_{ij}}{D_{n}}=\frac{D_{ij}}{1/3(D_{xx}+D_{yy}+D_{zz})},\;\;\text{with}\;\;i,j=x,y,z\;.$$ (12)

The mean diffusivity *D*_*n*_: = 1/3Tr(**D**) is a scalar denoting the measure of the total amount of diffusion inside a voxel and it is related to the inverse of the local tissue density. *D*_*n*_ does not contain any information on the anisotropicity of the region under consideration and it is thus very similar for both grey matter and white matter, whereas it is higher in the CSF region, where water diffusion is unconstrained. Thus, rewriting the diffusion tensor as **D** = *D*_*n*_ **T**, it is clear that the tensor **T** takes into account the preferential directions of the biased random movements of water molecules and, thus, it can also be used to describe the chemotactic motion. Fig 3(C) illustrates the components of tensor **T** on a slice clipped along the *xy*-plane in the middle point of the *z*-axis of the brain.

In Table 1, we summarize the values (or the ranges of values) for the different parameters appearing in the equation system Eqs (5) and (6). Let us now briefly discuss how we extrapolated the parameters in those cases in which the desired values were not explicitly found in literature. First of all, we dealt with the friction parameter *M*, that can be computed as the inverse of the hydraulic conductivity studied by [72], and we obtained values between 1377.9 and 4286.7 mm^−2^ Pa day. In order to estimate *ϵ* appearing in Eq (5), we referred to the measurements of the interstitial fluid pressure (IFP) *χ* and to the characteristic distance of interaction between cells, modeled by $\epsilon /\sqrt{\chi }$ and typically estimated to be in the order of the cell size [53]. In particular, [73] reported a IFP for healthy brain of 106.64 Pa, while [74] reported a mean IFP of 960 Pa for brain tumors by averaging the IFPs for meningiomas, glioblastomas and brain metastases. Consequently, knowing the values of *χ* and the size of a cell, which was experimentally estimated to be between 10 and 20 *μ*m [75, 76], it was possible to obtain the value of *ϵ*. The proliferation parameter *ν*_*c*_ varies between 24 h and 48 h [77, 78] for well oxygenated glioblastoma cells in vitro. However, since the proliferation rate relies significantly on the nutrient availability, also smaller value seems to be biologically admissible in the real condition and thus, in the case study presented in the following we hypothesized *ν*_*c*_ = 0.3 day^−1^. Regarding the threshold for cell death rate due to anoxia, its value is given in the range of 0.28–0.5 [75, 77, 79]. Accordingly, we used the value of 0.3 in the numerical simulations. The mean uptake rate can be extrapolated from biological measurements of the oxygen diffusion coefficient *D*_*n*_ in human brain and the distance, *l*_*n*_, covered by a molecule of oxygen before being uptake by a cancerous cell. The mean oxygen diffusion coefficient *D*_*n*_ in human brain reported in literature varies between 86.4 mm^2^day^−1^ [76, 77, 80] (which is also in agreement with the maximum mean diffusivity recorded in the DTI data in Fig 3(A)) and 156.5 mm^2^day^−1^ [79], while [77] estimated *l*_*n*_ ≈ 100 *μ*m. Thus, being $\delta_{n}=D_{n}/l_{n}^{2}$, an admissible range for *δ*_*n*_ is 8640–15650 day^−1^. The parameter *S*_*n*_ is quite difficult to be estimated from biological experiments, we referred to the value of 10^4^ day^−1^ reported in [53] for the human skin and we assume the same value for human brain. The physiological oxygen concentration *n*_*s*_ has been evaluated to be in the range 0.07–0.28 mM in [81]. Unfortunately, data on the chemotactic coefficient *k*_*n*_ of glioma cells in response to oxygen concentration are not present in literature and we had to refer to the typical chemotactic coefficient found for bacterial cells in response to glucose. Finally, [13] reported a Young’s Modulus *E* for both grey matter and white matter of about 694 Pa.

**Table 1:** Estimation of the biological parameters.

Parameter	Values	References
*ϕ*_*e*_, cell volume fraction at mechanical equilibrium	0.39	[82]
M, interphase friction	1377.9–4286.7 mm^−2^ Pa day	[72]
*χ*, IPF in healthy brain	106.64 Pa	[73]
*χ*, IPF in brain GBM	960 Pa	[74]
$\left(\epsilon /\sqrt{\chi }\right)$, GBM cell size	10–20 *μ*m	[75, 76]
*ν*_*c*_, GBM cell proliferation rate	0.5–1 day^−1^	[77, 78]
*δ*_*c*_, threshold for death cell rate due to anoxia	0.28–0.5	[75, 79]
*D*_*n*_, oxygen diffusion coefficient in brain	86.4 mm^2^day^−1^	[77]
*δ*_*n*_, oxygen consumption rate of the brain	8640 day^−1^	[77]
*S*_*n*_, blood tissue transfer rate of oxygen	10^4^ day^−1^	[53]
*n*_*s*_, oxygen concentration in brain vessels	0.07–0.28 nM	[81]
*l*_*n*_, oxygen penetration length	100 *μ*m	[77]
*k*_*n*_, chemotactic coefficient	1296 mm^2^ mM^−1^ day^−1^	[83]
E, Young’s modulus	694 Pa	[13]

Estimation of the biological model parameters from the experimental data on healthy brain tissue and glioblastoma.

## Results

### Sensitivity Analysis

**Fig 4:**
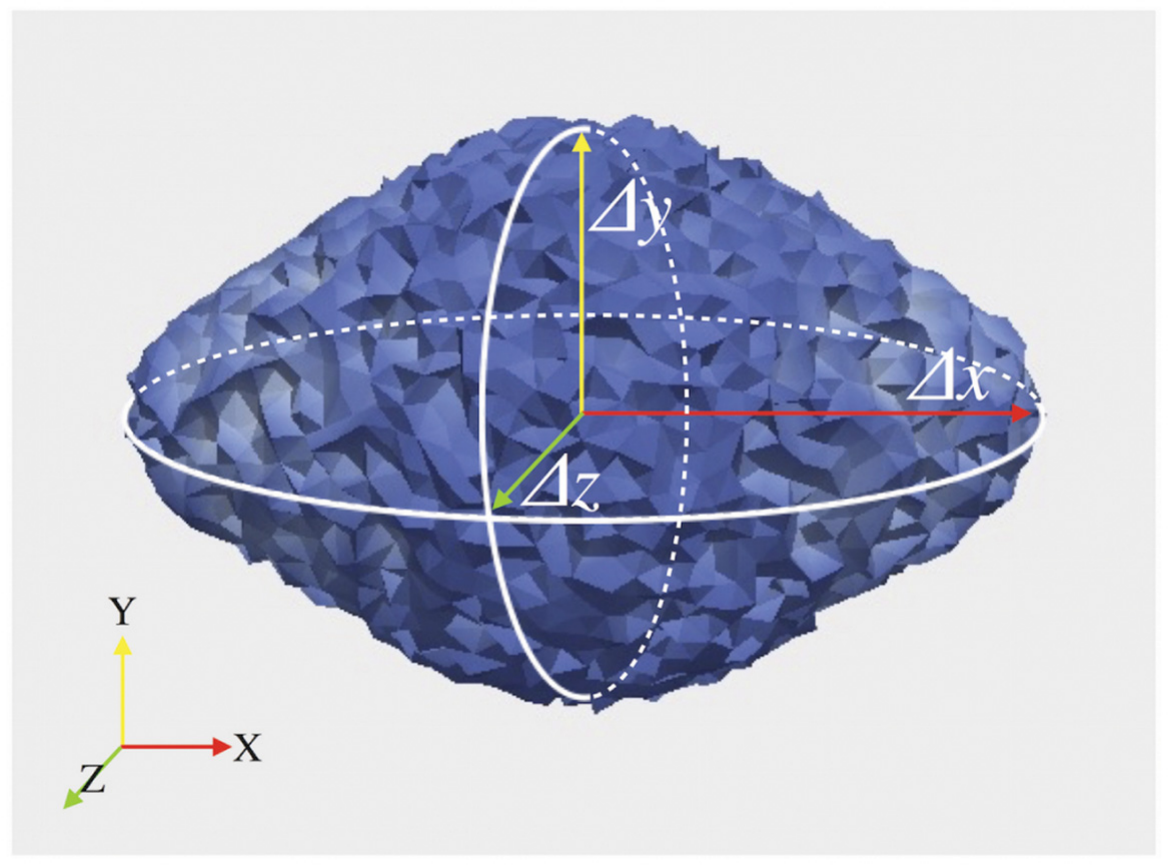
Tumor size parameters. The anisotropic growth of an initially spherical tumor is evaluated measuring the ratio between the major semi-axis and the minor ones of the grown tumor ellipsoid.

**Fig 5:**
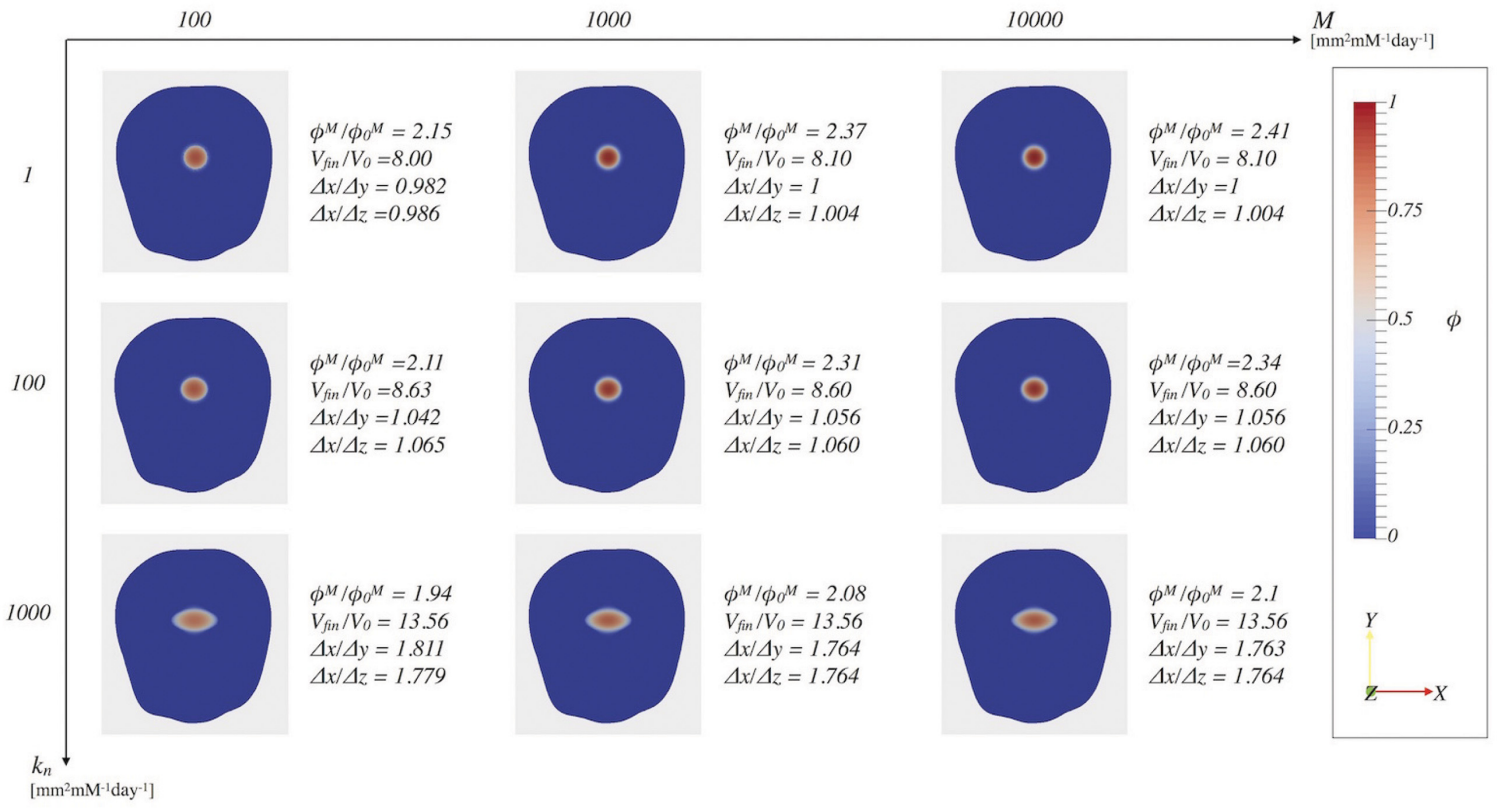
Sensitivity analysis of the parameters k_n_ and M. The influence of the parameters *k*_*n*_ and *M* on the cells volume fraction distribution at time *t* = 6 days is studied. The resulting tumor are characterized in terms of: the ratio between the maximum volume fraction at the final time, *ϕ* ^*M*^, and maximum initial volume fraction, $\phi_{0}^{M}$; the ratio between the final and the initial volume; the ratio between the major semi-axis, Δ*x*, and the two minor semi-axes, Δ*y* and Δ*z*, defined as in Fig 4.

**Fig 6:**
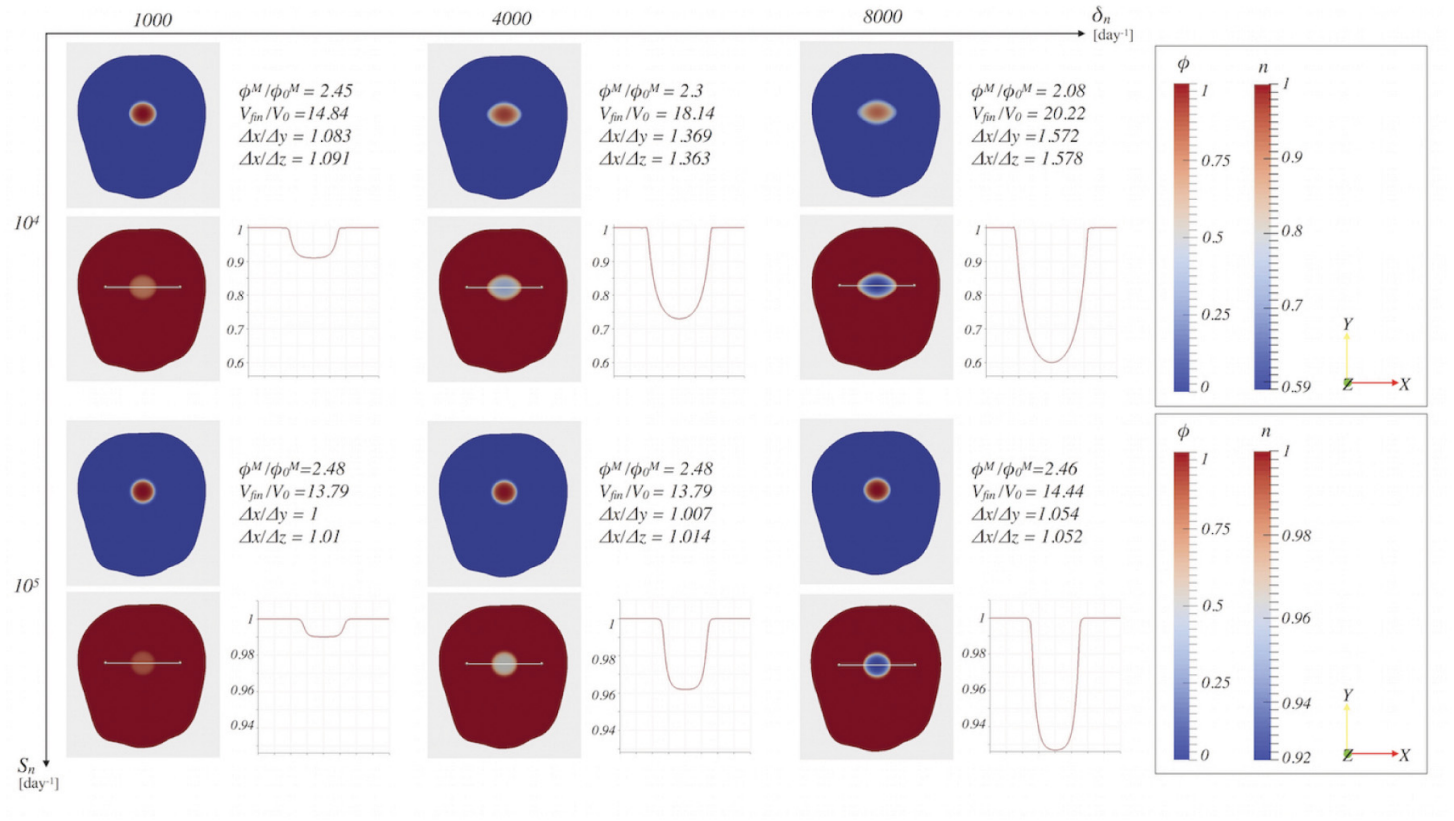
Sensitivity analysis of the parameters S_n_ and δ_n_. The influence of the parameters *S*_*n*_ and *δ*_*n*_ on the cell volume fraction and on the dimensionless nutrient concentration is reported at time *t* = 9 days.

In this section, we focus both on testing the physical soundness of the proposed model and on identifying which parameters in the model play a key role in the diffusion of nutrients and in the anisotropic growth of the tumor, evaluated measuring the ratio between the major semi-axis and the minor ones of the grown tumor ellipsoid (see Fig 4). Thus, we perform two sensitivity tests studying the combined effects of *M* and *k*_*n*_ on one hand, and of *S*_*n*_ and *δ*_*n*_ on the other, whilst keeping the other parameters fixed. The parameters *M* and *k*_*n*_ weight, respectively, the isotropic and the anisotropic expansion of the tumor, whereas the ratio between *S*_*n*_ and *δ*_*n*_ determines the oxygen availability in the tissues and, consequently, the tumor expansion through chemotactic motion. For the sake of simplicity, we locate a spherical tumor in the center of the brain and we impose the *x*-axis to be the preferential direction for oxygen diffusion and cell chemotaxis, setting *D*_*xx*_ = *D*_*n*_, *D*_*ii*_ = 0 for *i* = {*y*, *z*} and the off-diagonal components *D*_*ij*_ = 0. Fig 5 reports the *ϕ* distribution on the *xy*-plane at time *t* = 6^*th*^ day, for different values of the parameters *k*_*n*_ and *M*, whereas Fig 6 reports the *ϕ* and *n* distribution on the *xy*-plane at time *t* = 9^*th*^ day, for different values of the parameters *S*_*n*_ and *δ*_*n*_. The nutrient concentration has been normalized with respect to the physiological concentration *n*_*s*_, so that the numerical solution for *n* will range between 0 and 1. In all the simulations, we set *D*_*n*_ = 86.4 mm^2^day^−1^, *ν* = 1 day^−1^, *n*_*s*_ = 0.07 mM, *δ* = 0.3, *χ* = 900 Pa, *E* = 694 Pa and *ϕ*_*e*_ = 0.389.

Both in Figs 5 and 6, the grown tumor shape is analyzed in terms of the ratio between the maximum final cellular volume fraction over the maximum initial one, the ratio between the final and the initial volume of the tumor and the ratio between the major semi-axis of the tumor ellipsoid (Δ*x*) and the two smaller ones (Δ*y* and Δ*z*), which are good markers of the level of anisotropicity. The values of Δ*x*, Δ*y* and Δ*z* have been obtained defining the tumor ellipsoid as the region of the brain in which the cellular volume fraction is over a given threshold *ϵ*_*t*_ and computing the lengths of its semi-axes, as illustrated in Fig 4.

From the tumor data reported in Fig 5, we observe that the ratio of maximum cell volume fraction at the final and the initial time increases as *M* increases, while the ratio of the major semi-axis on the minor semi-axes and the total volume of the tumor ellipsoid are not significantly affected. Indeed, higher values of *M* inhibit the isotropic diffusive motion of cells (weighting the function *f*) and the repulsive interactions among them (weighting the term *ɛ* ^2^∇*ϕ*), and consequently, cancerous cells tend to accumulate (increased *ϕ* ^*M*^). Furthermore, the interface host/tumor gets sharper as *M* increases. Indeed, as observed also in [47], the ratio *ϵ* ^2^/*M* is related to the sharpness of the interface host/tumor. Regarding the chemotactic parameter, instead, it is possible to notice that, for small values of *k*_*n*_ (e.g. *k*_*n*_ = 1 mm^2^mM^−1^day^−1^), the tumor is almost spherical. On the contrary, as *k*_*n*_ increases the tumor acquires an ellipsoidal configuration, characterized by an increasingly bigger ratio between the longer and smaller semi-axes. Indeed, *k*_*n*_ weights the components of the tensor **T**: an increase of *k*_*n*_ has the effect of intensifying the movement of the cells along that preferential direction (i.e. the *x*-axis in the depicted cases). Consequently, considering a fixed proliferation rate of tumor cells, the maximum value *ϕ* ^*M*^ reached at a given time decreases, for increasing value of *k*_*n*_, due to the higher chemotactic response experienced by tumor cells, which is also represented by the increase of the total volume occupied by the tumor. Therefore we found that the parameter *M* affects the distribution of *ϕ* inside the tumor region and its maximum value, along with the smoothness of the tumor/host interface, whereas it does not affect tumor sizes at a given time step. On the other hand, the tumor isotropic/anisotropic expansion is strongly regulated by the chemotactic parameter *k*_*n*_.

The other two considered parameters, *S*_*n*_ and *δ*_*n*_ (see Fig 6), are primarily responsible of the nutrient spatio-temporal evolution and, as a consequence, of the evolution of the tumor fraction *ϕ*. Indeed, *S*_*n*_ is the parameter that regulates nutrients supply from blood vessels to tumor cells: thus, if its value is not high enough to overcome the nutrients consumption, regulated by the parameter *δ*_*n*_, the tumor does not receive enough nutrients to further expand. First of all, we observe that if the value of *S*_*n*_ increases, then the maximum value *ϕ* ^*M*^ reached inside the tumor region at a given time step increases while the total volume occupied by the tumor decreases. Conversely, considering the same value of *S*_*n*_ but increasing *δ*_*n*_, we observe that the ratio between *ϕ* ^*M*^ and $\phi_{0}^{M}$ decreases and the total tumor volume increases. Moreover, for high values of *S*_*n*_ and small values of *δ*_*n*_ the tumor grows almost spherically. To explain the behavior observed in tumor evolution, it is useful to look at the nutrient distribution inside the domain. As a matter of fact, the ratio between production and consumption of nutrients, i.e. *S*_*n*_/*δ*_*n*_, determines the minimum value *n*_*min*_ reached by the dimensionless nutrient concentration at a given time step and consequently the gradient of *n*. For the same value of *δ*_*n*_, it is found that as the production term governed by *S*_*n*_ decreases, *n*_*min*_ decreases too and, consequently, ∇*n*, which drives the chemotaxis, increases. Therefore in the case of a small *S*_*n*_, tumor cells proliferate less and move more, leading to a bigger but less populated (i.e. having a smaller *ϕ* ^*M*^) tumor region. At the same time, keeping fixed *S*_*n*_ and increasing *δ*_*n*_, *n*_*min*_ decreases and ∇*n* increases, leading to a bigger final tumor volume also in this case. Therefore, besides determining the spatio-temporal distribution of nutrients, both *S*_*n*_ and *δ*_*n*_ affect the expansion of the tumor, favoring the anisotropic growth in the case of low values of *S*_*n*_ and high values of *δ*_*n*_.

### Effect of Local Anisotropy in GBM: A Case Study

The sensitivity analysis allowed to understand the model behavior under different sets of parameters and it is essential in order to check the mathematical validity of the proposed approach. However it was performed under simplified conditions for the diffusivity tensor, therefore it is not suitable for clinical use. In the following, we integrate patient-specific radiological data in order to study the effects of the brain micro-structure on GBM evolution.

**Fig 7:**
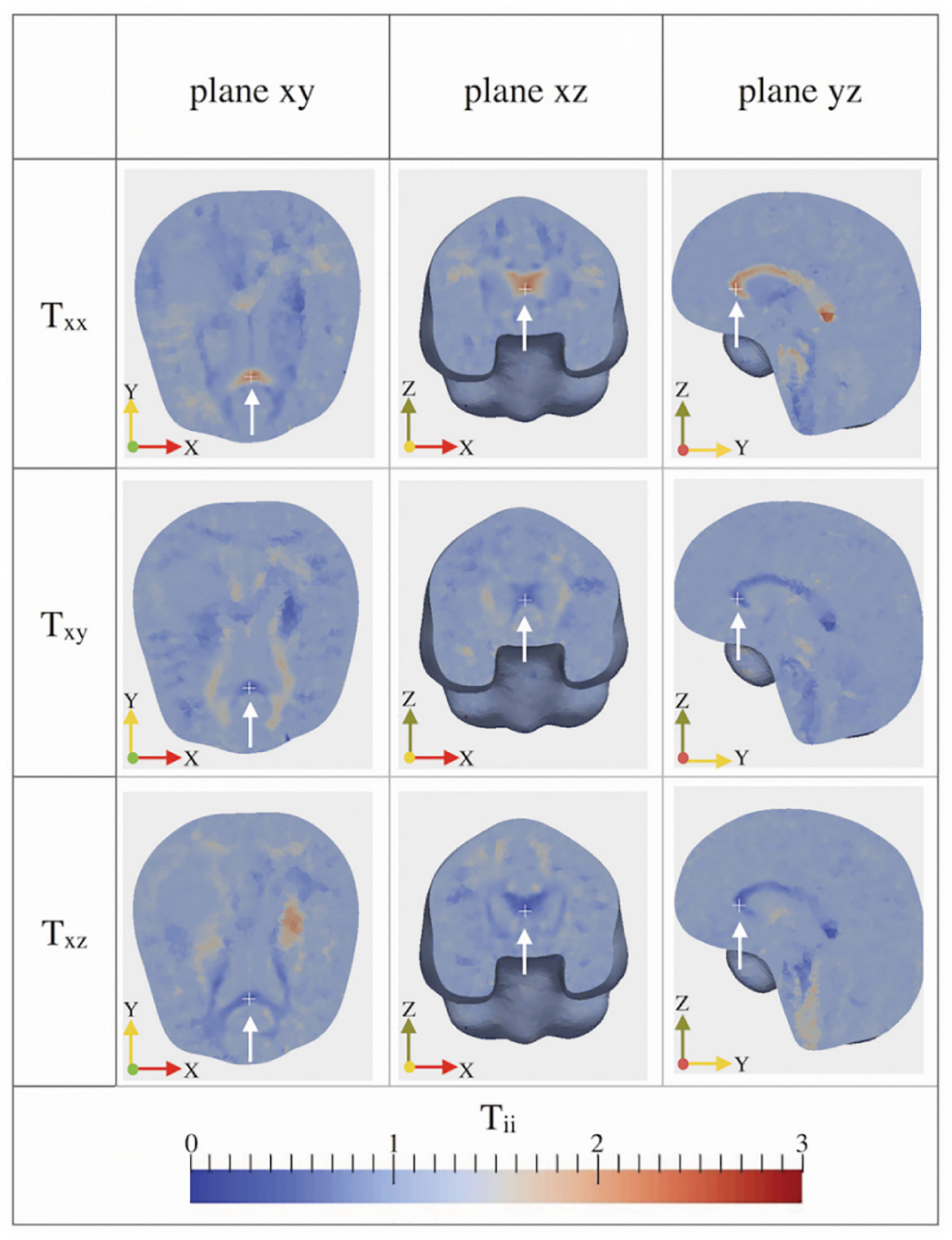
Diagonal components of the tensor T over the brain mesh cut along each plane. The components *T*_*ii*_ are represented over the brain mesh cut along the *xy*, *xz* and *yz* planes. The initial location of the virtual tumor, that corresponds to the corpus callosum, is indicated by a white cross.

We assume a virtual diagnosed tumor located in a brain region characterized by high anisotropy, such as the region occupied by the *corpus callosum* (i.e. between the lateral ventricles, above the thalamus and under the cerebrum). In Fig 7, we illustrate the tensor components *T*_*ii*_, with i = x, y, z, over a mesh clipped along each plane, and we indicate the tumor location with a white cross. Observing the collected snapshots reported in Fig 7, we highlight that, in the region of interest, *T*_*xx*_ is the component with the highest value: in fact it ranges between two and three, while *T*_*yy*_ and *T*_*zz*_ are close to zero. Consequently, the cancerous cells confined in that region will tend to move along the *x*-direction snd we expect that the tumor will grow anisotropically, losing its initial spherical shape.

**Fig 8:**
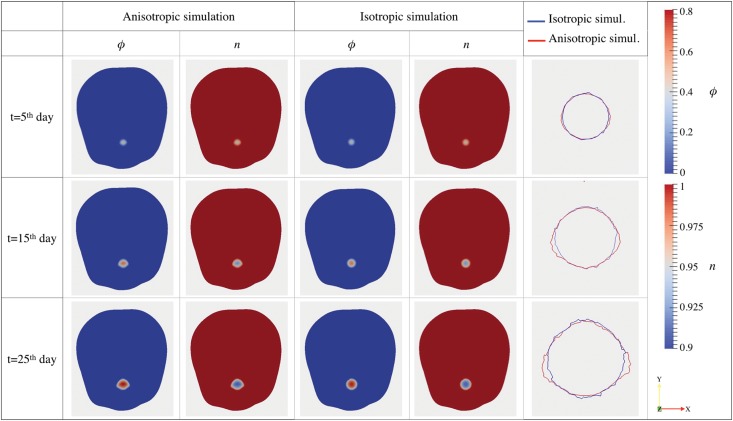
Comparison between anisotropic and isotropic growth. For both the anisotropic and the isotropic simulations, we report the tumor volume fraction distribution, the dimensionless nutrient concentration and the tumor contour plot at *t* = 5 day, *t* = 15 day and *t* = 25 day over the computational mesh cut along the *xy*-plane. In the anisotropic simulation the tensor **D** and **T** are the one reported in Fig 3(B)–3(C), respectively, whereas in the isotropic simulation we set **D** = *D*_*n*_ **I** and **T** = **I**.

The results obtained considering patient-specific **D** and **T** tensors (*anisotropic simulations*) are then compared in Fig 8 to the isotropic growth paths obtained in the case in which no information on the underlying brain structure is considered (*isotropic simulations*), i.e. setting **T** = **I** and **D** = *D*_*n*_ **I**, where *D*_*n*_ is defined as in Table 1 and **I** is the identity tensor. All the other parameters in the anisotropic and isotropic simulations are kept the same: *M* = 5000 mm^−2^ *Pa*, *S*_*n*_ = 10^4^ day^−1^, *δ*_*n*_ = 1000 day^−1^, *ν* = 0.25 day^−1^, *k*_*n*_ = 100 mm^2^ mM^−1^ day^−1^, *ϵ* = 0.02. We perform the simulation until the 25^*th*^ day after the *virtual* diagnosis.

Fig 8 reports the spatial distributions of *ϕ* and *n* over the computational mesh cut along the *xy*-plane at time steps *t* = 5, 15, 25 day, both for the anisotropic and the isotropic simulations. We observe that, in the anisotropic simulation, the expanding GBM mass loses its initial spherical shape and it assumes a configuration that reflects the structure of the tensor **T**, whereas in the isotropic simulation the glioblastoma maintains the spherical configuration. The maximum values reached by the cellular concentration at a given time step are comparable, with a *ϕ* ^*M*^ slightly higher in the anisotropic simulation.

For what concerns the dimension of the glioblastoma at the final time *t* = 25^*th*^ day, in the anisotropic simulation, we measure an extension along the preferential direction of motion (i.e. the *x*-axis) equal to Δ*x* = 10.6 mm, whereas in the other directions we have Δ*y* = 8.85 mm and Δ*z* = 8.4 mm. In the isotropic simulation, on the other hand, the final tumor area is almost perfectly spherical and smaller, being Δ*x* = 8.8 mm, Δ*y* = 8.95 mm and Δ*z* = 9.15 mm.

**Fig 9:**
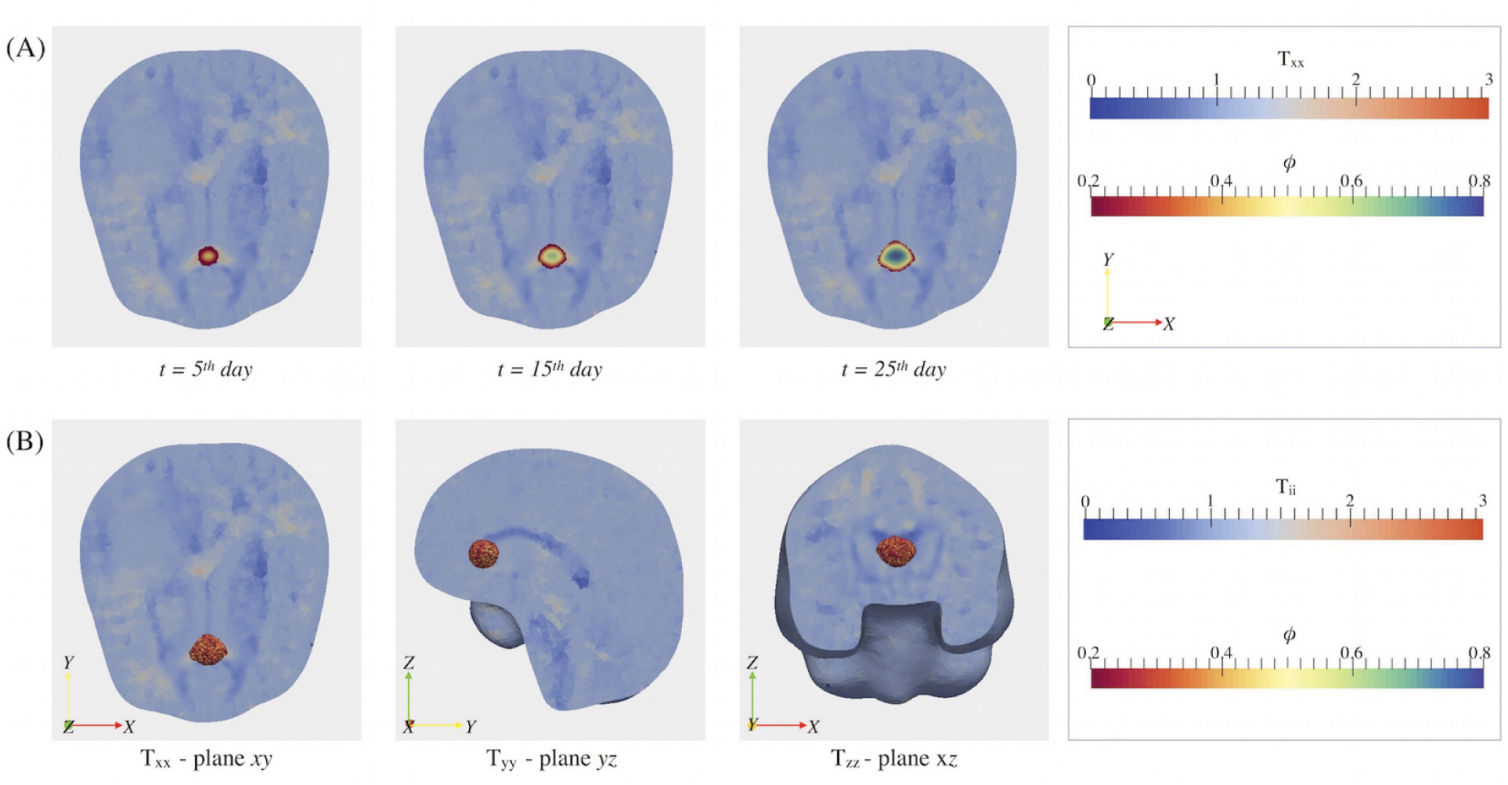
Influence of brain fibers’ alignment on tumor growth. (A) Tumor concentration plotted over the *T*_*xx*_ component (in transparency), at times *t* = 5 day, *t* = 15 day, *t* = 25 day: the cellular fraction shows an anisotropic distribution that follows the preferential direction determined by the *T*_*xx*_ component. (B) Tumor volume at *t* = 25 day overlapped to the maps of *T*_*xx*_ over the brain mesh cut along *xy* and *xz* planes and to the map of *T*_*zz*_ over the brain mesh cut along *xz*-plane: the glioblastoma assumes an elongated shape along the *x* direction, whereas it has a flat top in the *z*-direction, as *T*_*zz*_ is almost null there.

Plotting the projection of the tumor volume on the *xy*-plane in the anisotropic (red lines in Fig 8) an the isotropic case (blue lines in Fig 8), we notice that the mathematical model underestimates the total volume of the cancer if the anisotropic effect of fiber orientation are neglected. In fact, the resulting tumor shape in anisotropic simulations strongly affected by the underlying fiber orientation: plotting the thresholded *ϕ* at *t* = 5^*th*^, 15^*th*^, 25^*th*^ day overlapped to the *T*_*xx*_ components on a mesh cut along the plane *xy* (Fig 9(A)), we can notice that the tumor expansion follows the *x*-axis in the region in which *T*_*xx*_ is higher (red region) assuming a *conical* configuration. Observing the tumor volume at the final time step reported in Fig 9(B), it is clear that the tumor presents an elongated shape along the *x*-direction with a flat top due to the fact that *T*_*zz*_ is almost null in that region and thus the cells are not allowed to move along the *z*-direction.

## Discussion

In this work, we introduced a 3D continuous mechanical model, able to simulate the growth of a glioblastoma and the invasion of the surrounding tissue. In particular, we took into account patient-specific structural heterogeneity and anisotropicity and the evolution of nutrients inside the brain. Unlike other solid tumours, GBM consists of cells that can infiltrate deeply into the surrounding environment, so that the host/tumor interface is often not sharp and the density of GBM cells in the stroma at the tumor margin may not be detectable using existing imaging modalities. Thus, we considered a diffuse interface model for the GBM, in which no boundary conditions at the interface between the normal and the diseased region are required. Despite the diffused nature of the interface, the model is purely mechanical, substantially differing from reaction-diffusion models, such as the ones proposed in [20, 21, 28, 29, 30, 31, 34], since the equation governing the tumor evolution and motion are determined by thermodinamically-consistent mass and momentum balances. Furthermore, the motion of cells is not dictated by pure diffusion, but a chemotactic flux is introduced. This term not only represents the preferential motion of cells towards increasing concentration of nutrients, but it also reproduces cells motion along fibers directions, thanks to the introduction of the *tensor of preferential directions*, **T**.

The proposed model also differs from previous mixture models [47, 48, 77] and single-phase mechanical models [52] because it takes into account both the heterogeneity and the anisotropy of the brain tissues directly from DTI data.

Concerning the numerical simulations, we first created the computational mesh starting from a MR image of a patient affected by glioblastoma and, then, we extracted the heterogeneous and anisotropic components of the local diffusion tensor and of the tensor of preferential directions. Then, we discretized the resulting system of Eqs (5) and (6) using the finite elements method and we used the open-source software FEniCS [71] to develop the numerical codes.

Considering simplified conditions, we performed the sensitivity analysis of the model with respect to the biological parameters appearing in the governing equations to test their influence on the anisotropic growth of the tumor. We have found that both the chemotactic coefficient and the ratio between the nutrient supply and the consumption rate have a huge influence on the anisotropic growth of the tumor. The latter, indeed, determines not only the availability of nutrients in the environment but also cellular proliferation and migration, leading to an anisotropic cancer expansion in the case of low *S*_*n*_/*δ*_*n*_ ratios. The sensitivity analysis also demonstrated that the parameter *M* affects the distribution of *ϕ* inside the tumor region and its maximum value, in accordance with the smoothness of the tumor/host interface and without affecting the tumor size.

Finally, we tested the model in a biological meaningful situation, including patient-specific data collected from the DTI images of a patient. In the numerical simulations, we located the tumor in a region characterized by an appreciable anisotropy and we studied its development at different time steps, demonstrating that the tumor evolution is strongly influenced by the preferential direction identified by the tensor **T**. The obtained results have been also compared to the homogeneous and isotropic case, highlighting the importance of considering real anisotropic and patient-specific data in order to achieve a more truthful prediction of the tumor evolution and to possibly give indications for the clinical treatment of all those kinds of tumors, such as the glioblastoma, that grows in highly heterogeneous and structured environments.

The results presented in this work are promising and, to our knowledge, they represent the first implementation of a thermodynamically consistent continuous mechanical model on a 3D real geometry with the inclusion of patient specific data. In order to check its suitability for clinical use, the model should be tested under different biological situations (e.g. tumor resection and possible recurrences) and the numerical outcomes should be possibly compared to clinical data, obtained from the patient follow-up. Future refinements might either include anisotropic effects in the convective cellular velocity or introduce structural changes (e.g. fiber remodeling and mechanical properties alterations) due to tumor progression. Moreover, the proposed model considers a homogeneous distribution of blood vessels, through the nutrient supply term in the reaction-diffusion equation, thus neglecting the role of angiogenesis in GBM development, which is nowadays considered as a hallmark of the disease [84]. Accordingly, future refinements shall consider a patient-specific nutrient supply term, elaborating data on brain perfusion and vessel location, e.g. from Perfusion Weighted Imaging (PWI) techniques [85], such as the Dynamic Contrast Enhancement (DCE) MRI [86] and the Dynamic Susceptibility Contrast (DSC) MRI [86, 87].

Finally, the effect of medical therapy, such as chemotherapy or radiotherapy, on the evolution of the tumor should be introduced.

## Conclusion

In summary, we developed, analyzed and numerically simulated a diffuse interface binary mixture model able to describe GBM progression. The system of equations representing the spatio-temporal evolution of nutrients and tumor cells’ volume fraction was solved on a patient-specific 3D geometry, reconstructed from the MRI of a patient.

The model took into account not only biochemical factors such as nutrients availability but also mechanical interactions occurring between the local micro-environment and the tumor, which play a fundamental role in cancer progression and invasion. Moreover, for the first time in literature, we succeeded in introducing in a continuous mechanical model, the heterogeneity and the anisotropicity of the brain bundles from patient-specific DTI-images. The proposed approach represents a relevant improvement with respect to the current state-of-the-art for continuous mathematical models of GBM, i.e. the reaction-diffusion models developed in [21, 30, 31, 34, 51, 88], that do not provide any information on the stress that the expanding mass of tumour cells and associated inflammation exert on the healthy brain tissue. The results presented in this work are promising and make a step towards the ambitious purpose of providing tools to doctors in the treatment of this lethal tumor, allowing to test, along with standard treatments, also new therapeutic strategies based on the modulation of the mechanical stresses [89]. Finally, the proposed continuous mechanical model can be a perfect tool for defining a multiscale model for glioblastoma growth [90], as it potentially allows the upscale of information deriving from the smaller scales. Whilst subcellular and cellular mechanisms can be easily incorporated in GBM discrete/hybrid models [91] they cannot be incorporated in simple diffusion-reaction models, since they are controlled by mechanical and chemical interactions at the macroscopic scale. Therefore, future efforts will be devoted to the definition of a multiscale approach in order to combine the subcellular and the cellular discrete description into the macroscopic continuous representation of the whole process.

Indeed, only a multiscale and multidisciplinary approach combining clinical and radiological data with a mathematical model able to capture phenomena occurring at different scales, has the potential to foster our understanding on GBM evolution in every single patient throughout his/her oncological history, in order to target therapies in a patient-specific manner.
