# Correction: Towards the Personalized Treatment of Glioblastoma: Integrating Patient-Specific Clinical Data in a Continuous Mechanical Model

**DOI:** 10.1371/journal.pone.0143032

**Published:** 2015-11-10

**Authors:** Maria Cristina Colombo, Chiara Giverso, Elena Faggiano, Carlo Boffano, Francesco Acerbi, Pasquale Ciarletta

The following information is missing from the Funding section: This study was partly supported by European Commission funding through the FP7 program (grant 2566050).
